# Activation and Cell Death of Mouse Eosinophils in Response to Different Microenvironmental Stimuli

**DOI:** 10.3390/cells15060490

**Published:** 2026-03-10

**Authors:** Immaculeta Osuji, Nives Zimmermann

**Affiliations:** 1 Department of Pharmacology, Physiology and Neurobiology, University of Cincinnati College of Medicine, Cincinnati, OH 45267, USA; osujiia@mail.uc.edu; 2 Department of Pathology and Laboratory Medicine, University of Cincinnati College of Medicine, Cincinnati, OH 45267, USA; 3 Division of Allergy and Immunology, Cincinnati Children’s Hospital, Cincinnati, OH 45229, USA

**Keywords:** eosinophil, cell death, apoptosis, necrosis

## Abstract

In inflammatory states, eosinophils are exposed to stimuli leading to activation, increased survival, and/or different cell death subroutines, which have differing effects on tissue inflammation. The mechanisms of signal integration are poorly understood. In this manuscript, we investigated cell death types in response to stimuli mimicking the inflammatory microenvironment. Mouse bone marrow-derived eosinophils (BMDeos) were stimulated with cytokines, cell-cell interaction mimics, pathogen-associated molecular patterns (PAMP), and broad cell activation stimuli. Both PMA and crosslinking of CD95 (cCD95) induced cell death of BMDeos. However, cCD95-induced cell death was consistent with apoptosis, while activation with PMA lead to EETosis. Both stimuli lead to caspase 3 activation and increased total level of histone H3 citrullination, indicating that these outcomes are not able to discriminate between the two cell death types. Flow cytometry for annexinV/7AAD pattern at early time points, and morphologic assessment by immunofluorescence (for DNA, eosinophil granule protein and citH3) were the most reliable outcomes for distinguishing the cell death subtypes. While LPS alone did not decrease BMDeos viability, LPS in the presence of caspase inhibition (zVAD) caused delayed cell death, which did not conform to either of the two cell death types. Finally, LPS and LPS/zVAD led to an increased level of surface expression of CD274 (type 1 activation), while both cCD95 and PMA increased the surface expression of CD101 (type 2 activation). In summary, at least three different activation-associated cell death pathways are seen in BMDeos activated with microenvironment-mimicking stimuli. Crosslinking CD95 leads to type 2 activation and apoptotic cell death. PMA also leads to type 2 activation but EETosis-associated cell death. LPS and LPS/zVAD are associated with type 1 activation, and only LPS/zVAD lead to cell death via a subtype different from both apoptosis and EETosis.

## 1. Introduction

Eosinophils are multifunctional granulocytes derived from the bone marrow that comprise a minor fraction of circulating leukocytes under homeostatic conditions. Despite their relatively low abundance in peripheral blood, eosinophils play critical roles in both innate and adaptive immunity. Their accumulation in tissues characterizes a diverse group of inflammatory disorders known as eosinophil-associated diseases (EADs), encompassing allergic diseases (such as asthma and atopic dermatitis), helminthic infections, and primary eosinophilic syndromes including eosinophilic gastrointestinal disorders (EGIDs) and hypereosinophilic syndromes (HES) [1,2,3]. Compelling evidence from both murine models and human studies supports a pathogenic role for eosinophils in various disease contexts. Mouse models deficient in eosinophils such as ΔdblGATA, PHIL, and iPHIL strains, have been instrumental in delineating the detrimental functions of eosinophils in allergic airway inflammation, tissue fibrosis, and gastrointestinal inflammation [4,5,6,7]. In patients, eosinophil depleting therapies such as anti-IL-5 monoclonal antibodies (e.g., mepolizumab) have demonstrated efficacy in conditions including severe eosinophilic asthma and HES [8,9]. However, the benefit of such interventions is less pronounced in patients with eosinophilic esophagitis (EoE) [10], indicating that mere presence of eosinophils does not always signify their primary pathogenic role. Indeed, eosinophils have been implicated in tissue homeostasis, immunoregulation, and metabolic control [11,12,13], highlighting the need to distinguish pathogenic from homeostatic eosinophil phenotypes.

A common histopathological hallmark of eosinophil-mediated tissue damage is the presence of extracellular granules and granule proteins in affected tissues. These eosinophil mediators are released via degranulation, a key effector function of eosinophils, and contribute to both immune defense and tissue pathology [14,15]. One form of degranulation, termed cytolysis, represents both a degranulation method and a distinct form of cell death [16]. Historically, cell death was understood as dichotomous between apoptosis and necrosis, with apoptosis being a regulated, biochemically coordinated process not leading to inflammation, and necrosis being accidental and leading to inflammatory consequences. It was not until the early 2000s that this long-standing notion of necrosis as an exclusively accidental process began to be challenged. Research revealed that certain forms of necrosis were, in fact, executed through defined signaling pathways, a concept now referred to as regulated necrosis [17]. As the field expands and overlap between certain features of different cell death subroutines are identified, the Nomenclature Committee on Cell Death provides standardized guidelines for morphological, biochemical and functional classification of cell death types focusing on mechanistic and essential aspects of each specific cell death process [17].

Eosinophil cell death has been increasingly recognized as a regulated process. Specifically in human eosinophils, in vitro studies have shown multiple death pathways including apoptosis [18] and regulated necrosis pathways such as necroptosis [19,20] and EETosis [21,22]. However, not all regulated necrosis in human eosinophils fits canonical definitions [17], especially in co-activated cells [23] and a ferroptosis-like process [24]. Tissue studies in diseases such as EoE, HES, and asthma reveal substantial evidence of eosinophil cytolysis and other forms of regulated necrosis, including markers such as phosphorylated MLKL and extracellular DNA traps [20,25,26], linking eosinophil death to inflammatory pathology in vivo. Mouse eosinophils, though less well characterized in this regard, have also been shown to undergo regulated necrosis upon exposure to specific stimuli including MPT-driven necrosis [27], pyroptosis [28] and a ferroptosis-like process [24]. However, a comprehensive framework for distinguishing apoptosis from various forms of regulated necrosis in eosinophils remains elusive. This gap is further compounded by the complexity of tissue specific microenvironments, where eosinophils are exposed to diverse combinations of immunologic, microbial, and stromal signals that influence their mode of death. The general premise is that understanding the nuances of eosinophil death will provide a framework for delineating between homeostatic and pathogenic eosinophils in various tissues and disease contexts.

In this study we assessed how distinct microenvironmental stimuli modulate mouse eosinophil survival and type of cell death in vitro. Our studies show at least three distinct regulated cell death pathways in eosinophils, exemplified by crosslinked CD95 (apoptosis), PMA (EETosis) and LPS/zVAD activation. Furthermore, our data show association of type 2 activation with at least 2 of the cell death pathways (apoptosis and EETosis), while type 1 activation led to cell death only when caspase activity was inhibited. Together, these findings deepen our understanding of eosinophil activation and cell death subroutines.

## 2. Materials and Methods

### 2.1. Isolation and Ex Vivo Culture of Mouse Bone Marrow-Derived Eosinophils (BMDeos)

The ex vivo culture of BMDeos was performed using the Dyer method [29]. Briefly, bone marrow cells were flushed from mouse femurs and tibiae using warm culture media. Red blood cells were lysed with RBC lysis buffer (Fisher cat#50-112-9751). Cells were cultured at 1 × 10^6^/mL in complete BMDeos media, which contains RPMI 1640 with L-glutamine (Thermo Fisher Scientific, Waltham, MA, USA cat#22-400-105) containing 20% fetal bovine serum (Thermo Fisher Scientific cat#MT35011CV), penicillin/streptomycin (Thermo Fisher Scientific cat#15140122), 2 mM glutamine (Invitrogen, Carlsbad, CA, USA), 25 mM HEPES (Gibco, Grand Island, NY, USA, 15630080), nonessential amino acids (Gibco, 11140050), Sodium Pyruvate (Gibco, 11360070), and β-mercaptoethanol (Thermo Fisher Scientific cat#125472500). Culture was supplemented with stem cell factor (mSCF, PeproTech, Cranbury, NJ, USA; 250-03) and fms like tyrosine kinase 3 (FLT3) ligand (mFLT-3L, PeproTech; 250-31), each at a 100 ng/mL final concentration added every other day for the first 4 days of culture. Beginning from day 4, cells were cultured in fresh medium containing recombinant mouse IL-5 (mIL-5, 10 ng/mL; PeproTech; 215-15). Culture progress was monitored by cell count, morphology, and flow cytometry. On days 13–15, the cells were collected for use in subsequent experiments.

### 2.2. Cell Stimulation

Cells were transferred into stimulation wells at 1 × 10^6^ cells/mL in complete BMDoes media unless otherwise stated, and incubated for 15 min–72 h (exact time given in text and figure legends) with the following stimuli: PMA (2 ng/mL, 15 ng/mL; Millipore Sigma, Burlington, MA, USA, cat#P8139), anti-CD95 (100 ng/mL; BD pharmingen, Milpitas, CA, USA, cat#554255), protein A/G (2 μg/mL, Thermo Fisher Scientific ref#21186), IL5 (10 ng/mL), LPS (0–100 ng/mL; Millipore Sigma cat#L2880), and zVAD (20 μM; Promega, Madison, WA, USA, cat#G723B).

### 2.3. Flow Cytometry

Flow cytometry was used to assess BMDeos development during culture, assessment of cell death, and activation state. Details are presented in MIflowcyt format in the Supplementary Data. Briefly, for assessing eosinophil maturation, single cell suspensions were Fc-blocked using anti-mouse CD16/CD32 antibody (Thermo Fisher Scientific) for 10 min on ice in FACS buffer followed by surface staining with Siglec-F-PE (Biolegend, San Diego, CA, USA, cat#155506), CCR3-BV421 (Biolegend cat#144517), and rat anti-mouse CD45-APCcy7 (BD Pharmingen cat#1046748) for 30 min on ice, which were washed and resuspended in flow buffer with 7AAD (Thermo Fisher Scientific cat#006993-50) and were incubated at room temperature for 15 min prior to flow analysis. For assessment of cell death, single cell suspensions were Fc-blocked, stained with Siglec-F-PE (Biolegend cat#155506) and CCR3-BV421 (Biolegend cat#144517), washed, and incubated for 15 min at RT with Annexin V (BD Biosciences, San Diego, CA, USA, Horizon™ BV605 Annexin V cat#563974) and 7-AAD Viability Staining Solution (Thermo Fisher Scientific cat#00-6993-50) in Annexin V buffer (BD Biosciences, cat#51-66121E). For activated caspase 3 staining, intracellular staining of activated caspase 3 was carried out per BD Pharmingen FITC active caspase Apoptosis kit (BD Biosciences, cat#550480). After treatment, cells were fixed and permeabilized with BD cytofix/cytoperm solution, washed and incubated with FITC-conjugated monoclonal rabbit anti-active Caspase-3 antibody in perm/wash solution. Cell activation status was assessed using CD274-APC (Biolegend cat#124311) and CD101-AlexaFluor700 (Thermo Fisher Scientific, cat#56-1011-82). Flow cytometry acquisition was performed on a BD FACSCanto 3 flow cytometer or LSR Fortessa (BD Biosciences, San Diego, CA, USA) and analyzed with FlowJoV10.10.0 Software (Tree Star).

### 2.4. Cytomorphology

Following stimulation, approximately 2 × 10^5^ cells were cytospun onto a glass slide, and stained using kwik-diff kit (Fisher Scientific cat#9990702). Cells were assessed morphologically for features of apoptosis (such as condensed chromatin and pyknotic nuclei) and necrosis (such as karyorrhectic nuclei, cell rupture and fragmentation).

### 2.5. Cell-Free DNA Measurement

Supernatant from cells following treatment were analyzed for dsDNA using Quant-iT PicoGreen dsDNA kit (Thermo Fisher Scientific, cat#P7589) according to the manufacturer’s specification. Briefly, 1 × 10^6^ BMDeos were stimulated with the indicated stimuli at the noted time points. After stimulation, supernatants were collected and Picogreen fluorescence was measured in 96-well fluorescence microplates (Thermo Fisher Scientific, cat#M33089) using GloMax-Multi Detection System microplate reader (Promega, Madison, WI, USA).

### 2.6. Immunofluorescence

The cells were stimulated as described above in ibidi µ-Slide 8 Well (Cat. 80806) at a density of 2 × 10^5^ cells/well. After stimulation, the plates were spun at 15 °C, 600 *g* for 2 min (Avanti J-15 series centrifuge, Beckman Coulter, Brea, CA, USA). Medium was carefully removed from each well and cells were washed with cold PBS and fixed with 10% formalin at room temperature (RT) for 10 min, followed by 3 washes with cold PBS. After fixation, cells were permeabilized with perforation buffer (0.5% Triton-X-100 in PBS) for 10 min at room temperature and then washed with PBS. Samples were incubated in blocking buffer (1% BSA + 0.2% Triton X 100 in PBS) for 30 min at RT, followed by overnight incubation with the primary antibodies (rat anti-mouse MBP clone: MT2-14.7.3, received from Elizabeth Jacobsen, Ph.D., Mayo clinic; rabbit recombinant multiclonal antibody to citrullinated histone H3 (citH3, Abcam cat#AB281584, Cambridge, UK)) at 4 °C, followed by respective secondary antibodies (goat anti-rat: Alexa Fluor 594, cat#A48264; donkey anti-rabbit: Alexa Fluor 488 cat#A32790). Cells were allowed to incubate for 30 min at RT and washed before mounting with ibidi Mounting Medium with DAPI (50011, ibidi, Fitchburg, WI, USA). Images were captured with TiE inverted SpectraX microscope (Nikon, Melville, NY, USA) at 40× objective and automatically analyzed using Nikon GA elements. In the software, total citH3 identified all citH3 staining with sizes ranging from 1.85–414.27 μm and “growing objects”. “Small” citH3 is defined as having a size range of 1.85–5.50 μm, “big” has a range of 5.51–12.29 μm, and netcitH3 has a size range of 12.30–414.27 μm and includes “growing objects” (net) (for details of automated analysis, please see Supplemental Figure S1).

### 2.7. Western Blotting

After cell stimulation, plates were spun at 400 *g*, washed with cold PBS and resuspended in 500 μl of Pierce RIPA buffer (Thermo Fisher Scientific cat#8990) supplemented with Halt protease and phosphatase inhibitor (Thermo Fisher Scientific cat#1861280) and incubated on ice for 15 min. Lysates were centrifuged at 13000 *g*, and supernatant saved. Protein concentration was determined by Pierce BCA Protein Assay Kit (Thermo Fisher Scientific) according to the manufacturer’s instructions. Lysates were incubated with LDS sample buffer and DTT, and heated at 70 °C for 10 min. Samples were loaded on 4–12% Bis-Tris Mini Protein Gel and transferred to nitrocellulose membrane. Membranes were blocked in 5% low fat milk for 60 min at room temperature, incubated overnight at 4 °C with primary antibodies (Cell signaling, Danvers, MA, USA, caspase 3 cat#9662S; 1:1000 and GAPDH cat#2118L; 1:5000) and 1 h at room temperature for the secondary antibody (GtxRb IgG F(ab’)2 HRP; Millipore sigma; AQ132P; 1:2000). Images were taken on Chemidoc imaging system version 2.4.0.03 (BioRad, Hercules, CA, USA) and quantified using ImageJ V1.52a.

## 3. Results

### 3.1. Eosinophils Undergo Different Patterns of Cell Death

**Figure 1:**
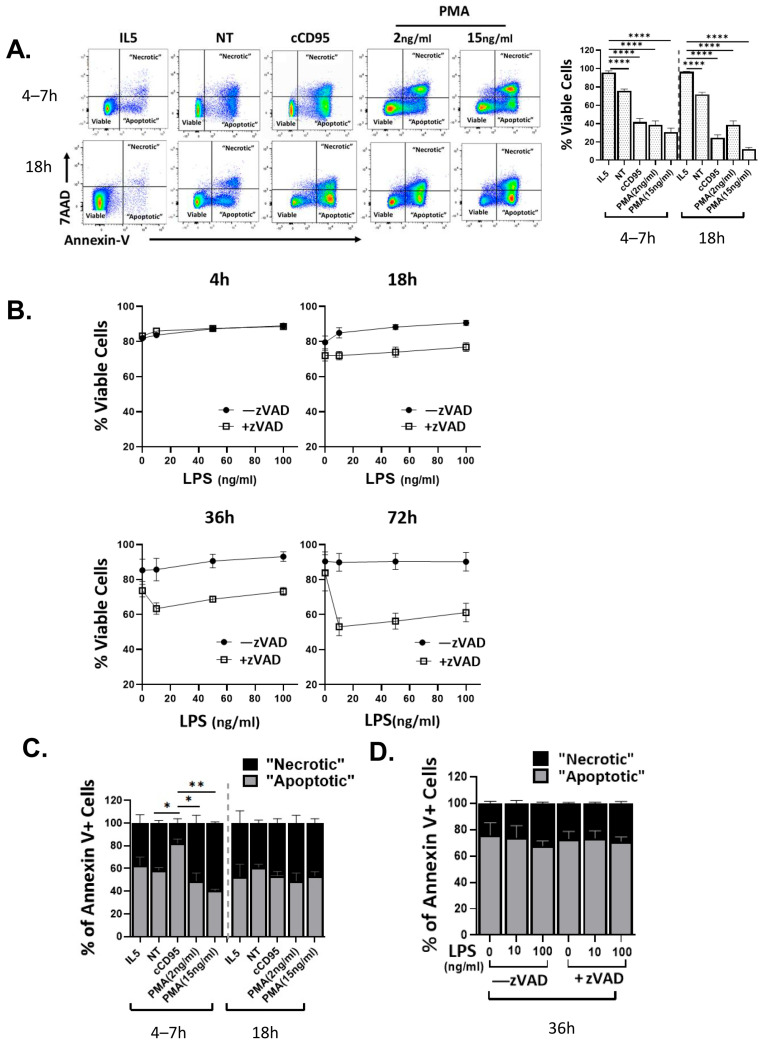
Eosinophil cell death in response to physiologic and pharmacologic stimuli as assessed by annexinV/7AAD flow cytometry. BMDeos were incubated with IL-5 (10 ng/mL), anti-CD95 crosslinked with protein A/G (cCD95, 100 ng/mL, 2 μg/mL, respectively), PMA (2–15 ng/mL), LPS (10–100 ng/mL) with and without zVAD (20 μM), and no treatment (NT) for indicated times (4–72 h). Cells were analyzed by flow cytometry, and defined as viable: annexin-V/7AAD-double negative; “apoptotic”: annexin V-positive/7AAD negative; and “necrotic”: annexin V/7AAD-positive. (**A**,**B**) Representative dot plots and quantification of % viable cells among cells treated with the shown stimuli. (**C**,**D**), % of “apoptotic” and “necrotic” cells among all dying cells (all annexin V-positive) are shown. Data are presented as mean ± SEM of 2–18 biologic replicates (individual cell cultures). Statistical analysis was performed using two-way ANOVA followed by pairwise comparisons using Dunnett’s multiple comparisons: * *p* < 0.05; ** *p* < 0.01; **** *p* < 0.0001.

In order to screen for different types of cell death in eosinophils, we stimulated mouse bone marrow derived eosinophils (BMDeos) with stimuli that mimic microenvironmental conditions including type 2 inflammation (e.g., IL-5) [30], cell–cell interaction (e.g., crosslinking of the Fas receptor, cCD95, mimicking the physiologically important FasL-induced pathway), broad cell activation stimulus (e.g., activation by protein kinase C activator phorbol 12-myristate 13-acetate (PMA), which mimics activation by receptor tyrosine kinases and/or G-protein coupled receptors) [31], and pattern recognition receptors, such as LPS, with and without caspase inhibition by zVAD (which mimics survival signaling by cytokines such as IL-5) [32]. We first assessed cell viability and death by flow cytometry for annexin V binding and membrane permeability (7AAD entry). Annexin V binds to phosphatidyl serines, which are normally present only on the inner leaflet of plasma membranes, but are exposed by either lack of ATP to power the enzyme flipase (as seen early in apoptosis), or loss of membrane integrity (as seen late in apoptosis and other cell death mechanisms). 7AAD enters cells that have lost membrane integrity. Thus, by analyzing the pattern of annexinV and 7AAD staining over time, one can begin to elucidate cell death types. As has been reported previously, the presence of IL-5 was required for optimal maintenance of eosinophil viability, in that IL-5 withdrawal (no treatment, NT) decreased proportion of viable cells (defined as annexinV− 7AAD−). Similarly, CD95 crosslinking and PMA, caused a decrease in viable cells in a time-dependent manner (Figure 1A). LPS alone maintained viability, while LPS in the presence of zVAD caused decreased cell death at later time points than PMA and cCD95 (Figure 1B).

We next assessed the proportion of cells in the “apoptotic” (defined as annexinV+ 7AAD−) versus “necrotic” (annexinV+ 7AAD+) state among all annexinV+ cells. We noted differential response to stimuli in terms of cell death state progression. For instance, IL-5 withdrawal and CD95 crosslinking caused eosinophils to largely transition through the “apoptotic” state at early time point to “necrotic” state at the later time point. In contrast, PMA-induced cell death shows a direct entry into the “necrotic” state already at the early time point (Figure 1C). When LPS/zVAD treated cells were assessed at the 36 h time point (earliest time of significant separation between LPS/zVAD and LPS or zVAD alone-treated cells), the majority of cells were in the “apoptotic” state (Figure 1D).

**Figure 2:**
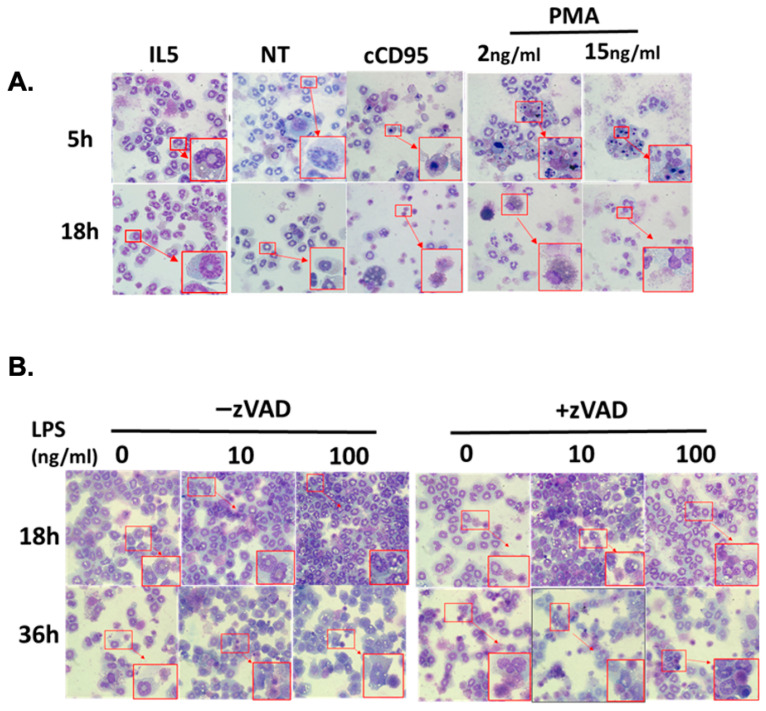
Cytology of eosinophils following treatment with physiologic and pharmacologic stimuli. (**A**) Representative images of cell morphology following treatment with IL-5 (10 ng/mL), anti-CD95 crosslinked with protein A/G (cCD95, 100 ng/mL, 2 μg/mL, respectively), PMA (2–15 ng/mL), and no treatment (NT) for 5 and 18 h. (**B**) Representative images of cell morphology following treatment with LPS (10–100 ng/mL) with and without zVAD (20 μM) for 18 and 36 h. Inset figure shows enlarged detail of the small red square in original figure.

One limitation of the flow cytometry-based approach for cell death assessment is that once cells are no longer intact, they are “gated out” and thus not captured by the analysis. Furthermore, cells are prevented from clumping through filtration and active exclusion of cell–cell association via doublet elimination. Since previous studies have shown eosinophil aggregation with activation [33,34], we assessed cell death type by a complementary method that does not exclude cell aggregates and non-intact cells. Specifically, we assessed the morphology of cells on cytospin preparations. Cytologic morphology revealed intact nuclear shape and cytoplasmic granularity consistent with mature eosinophils, with no significant difference observed with or without IL5 at the indicated times (Figure 2A). At the early time point, crosslinking CD95 showed a more apoptotic phenotype including condensed chromatin and pyknotic nuclei. PMA treatment induced noticeable aggregation of cells and cell remnants, with many cells showing nuclear fragmentation. At the later time point, most of the cells in both cCD95 and PMA treatment show features of necrosis such as karyorrhectic nuclei and/or ruptured or fragmented cells (Figure 2A). LPS with or without zVAD showed a distinctly different morphology. At 18 h, there was cytoplasmic vacuolation. By 36 h, while the vacuolization remained, a subset of cells showed nuclear pyknosis and budding, more pronounced in the presence of zVAD (Figure 2B).

In summary, these data show that multiple stimuli seen in the inflammatory microenvironment effect eosinophil viability, and that cells undergo different patterns of cell death, suggestive of different biochemical pathways.

### 3.2. Activated Caspase-3

**Figure 3:**
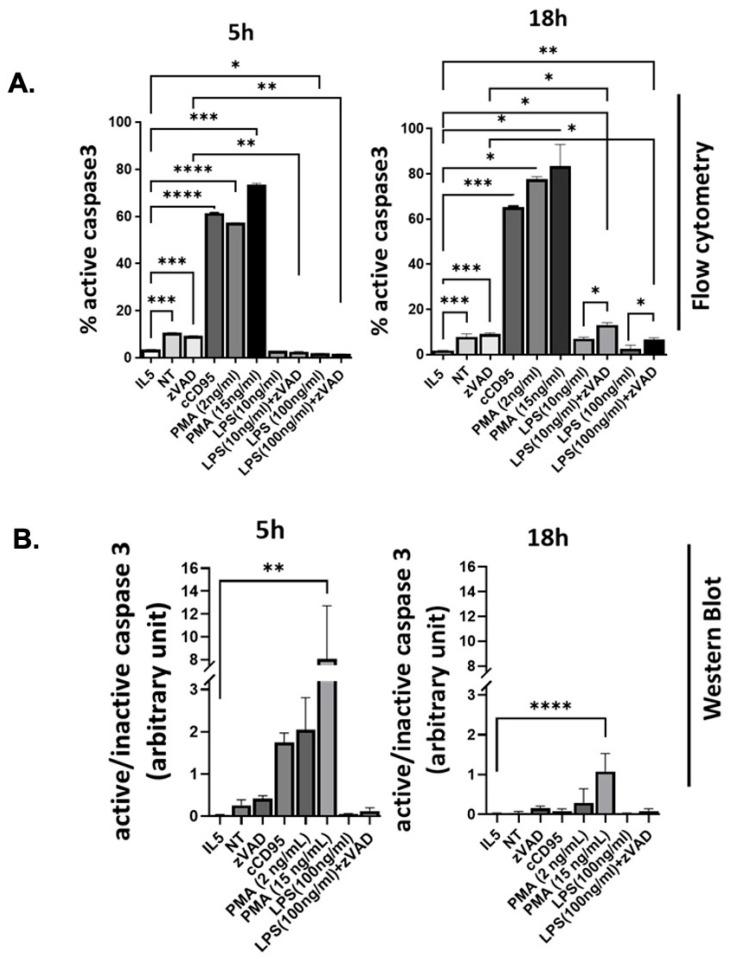
Detection of activated caspase-3 in stimulated BMDeos. BMDeos were stimulated with IL-5 (10 ng/mL), CD95 crosslinked with protein A/G (100 ng/mL and 2 μg/mL respectively), PMA (2 ng/mL and 15 ng/mL), LPS (100 ng/mL) with and without zVAD (20 μM), and unstimulated (NT) for 5 h or 18 h. (**A**) Cytoplasmic detection of activated caspase-3 by flow cytometry. Data are mean ± SD (*n* = 3 technical replicates) from a representative of 3 (5 h) and 6 (18 h) experiments. (**B**) Western blot analysis quantification of activated caspase-3 in whole cell lysates. Densitometric analysis of the cleaved caspase-3 band (~19 kDa) was normalized to inactive caspase-3 (~30 kDa) and is shown as mean ± SEM from 2–3 independent experiments. Statistical significance was determined using one-way ANOVA with pairwise comparisons using Sidak’s multiple comparisons test. * *p* < 0.05; ** *p* < 0.01; *** *p* < 0.001; **** *p* < 0.0001.

Activation of caspase-3 is a hallmark of intrinsic and extrinsic apoptosis. Thus, to test whether the apoptotic machinery is involved in the different cell death patterns, we assessed levels of activated caspase-3 in stimulated BMDeos. We first used flow cytometry as a more quantitative approach to assessing proportion of cells with activated caspase-3. The level of activated caspase-3 was lowest in IL-5 treated eosinophils and was slightly increased with IL-5 withdrawal (NT) at levels comparable at 5 and 18 h. Crosslinked CD95 and PMA caused a significant increase in levels of activated caspase-3, at levels comparable at 5 and 18 h (Figure 3A). There was a slight but statistically significant increase in proportion of cells with activated caspase 3 observed at 18 h time point by flow cytometry in LPS/zVAD stimulated cells, but not in ones stimulated with LPS alone (Figure 3A).

Since flow cytometry used only cells in suspension and with retained cell integrity, it might miss any activated cells that have adhered to the plate, and/or cells that have disintegrated to the point of exclusion as debris on FSC/SSC by flow cytometry. Thus, we used an alternative approach of assessing activated caspase-3 by Western blotting. Similar to flow cytometry, IL5-treated cells did not show activated caspase-3, while those without IL-5 (NT) showed low-level activation of caspase-3, especially at the 5 h time point. Similarly, crosslinked CD95 and PMA showed a greater level of activated caspase-3 at 5 h than 18 h, with only PMA reaching statistical significance (Figure 3B). Neither LPS alone nor LPS/zVAD showed statistically significant caspase 3 activation at 5 or 18 h by Western blotting (Figure 3B).

In summary, caspase-3 is activated by multiple stimuli, albeit at different levels and time points. Thus, these findings indicate that caspase3 is activated by multiple stimuli including those that are not classically associated with apoptosis, and is thus not unique to this cell death type.

### 3.3. Characterization of Regulated Necrosis/EETosis

There is no single gold standard for EETosis characterization. Accepted features include cell cytolysis, release of cell-free granules/granule proteins as well as release of DNA. Thus, we assessed the cardinal features of EETosis in stimulated BMDeos.

**Figure 4:**
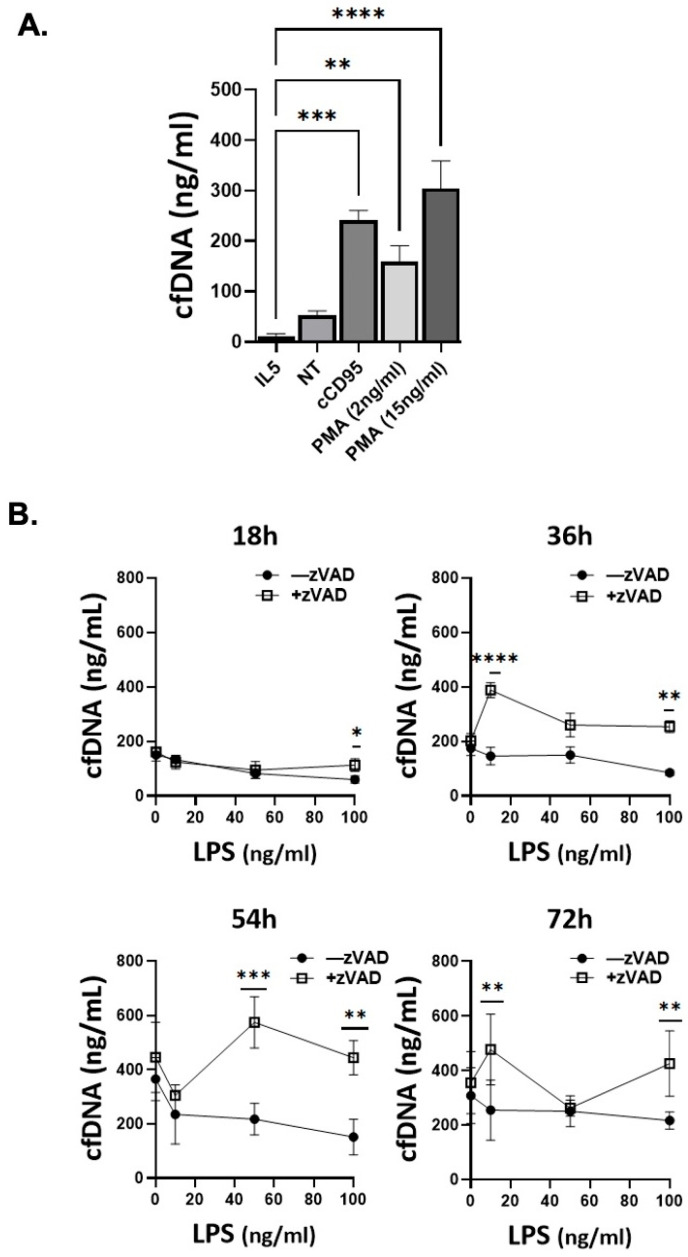
Release of cell free DNA (cfDNA) from BMDeos in varying cell death conditions. (**A**) BMDeos were stimulated with IL-5 (10 ng/mL), CD95 crosslinked with protein A/G (100 ng/mL, 2 μg/mL, respectively), PMA (2 ng/mL and 15 ng/mL), and unstimulated (NT) for 18 h, and cfDNA was measured in the cell supernatant. Data shown are mean ± SEM from three independent experiments. Statistical significance was determined using one-way ANOVA with pairwise comparisons using Bonferroni’s multiple comparisons test. ** *p* < 0.01; *** *p* < 0.001; **** *p* < 0.0001 (**B**) BMDeos were treated with LPS (10–100 ng/mL) with and without zVAD (20 μM) for 18–72 h. Data shown are mean ± SEM from four independent experiments. Statistical significance was determined using repeated measures one-way ANOVA with pairwise comparisons using Sidak’s multiple comparisons test. * *p* < 0.05; ** *p* < 0.01; *** *p* < 0.001 **** *p* < 0.0001.

In order to assess whether cell death induced by the different stimuli leads to disintegration of cell integrity with release of DNA, we assessed for cell free DNA (cfDNA) in the supernatants of cells stimulated with multiple stimuli at optimal doses, as established in the experiments above. While at 1 and 5 h there was no reproducible increase in cfDNA, at 18 h there was significantly increased cfDNA in the supernatant of cells stimulated with crosslinked CD95, and PMA. In contrast, cell-survival inducing stimuli such as IL5 showed no increase in the level of released cfDNA (Figure 4A). LPS/zVAD induced the release of cell-free DNA at later time points (36–54 h) while LPS alone showed no significant difference in the level of released cfDNA (Figure 4B). In summary, cfDNA release occurred with multiple cell death-inducing stimuli, and was not able to distinguish different types of cell death.

**Figure 5:**
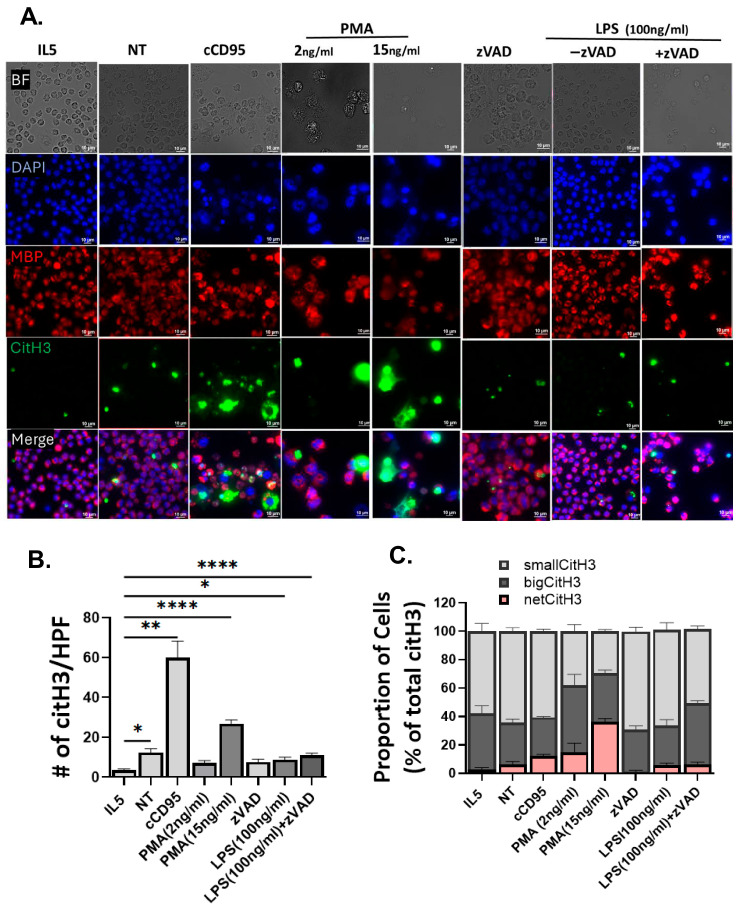
Assessment of eosinophil cell death type by immunofluorescence. BMDeos were incubated with IL5 (10 ng/mL), anti-CD95 crosslinked with protein-A/G (100 ng/mL and 2 μg/mL respectively), PMA (15 ng/mL), LPS (100 ng/mL) with and without zVAD (20 μM), or no treatment (NT) for 18 h. (**A**) Representative high magnification images of cells stained for DNA (DAPI, blue), citH3 (green), and eosinophil granule protein (MBP, red) are shown. Scale bar = 10 μm. (**B**,**C**) Automated citH3+ cell quantitation and assessment of size of citH3 signal is assessed. Small citH3 = 1.85–5.5 μm (light gray bar), big citH3 = 5.51–12.29 μm (dark gray), net citH3 = 12.3–414.27 μm (orange bar). Data shown are mean ± SEM from four independent experiments. Statistical analysis by one-way ANOVA followed by Dunnett’s multiple comparison test. * *p* < 0.05, ** *p* < 0.01, **** *p* < 0.0001.

Since measurement of cell-free DNA in the supernatant may miss DNA released but still associated with cells or bound to plastic, and to visualize the association of DNA and eosinophil granule proteins, we used immunofluorescent microscopy. Untreated cells (and those treated with IL-5) showed DNA in the nucleus and granule protein MBP in the cytoplasm of intact cells. When cells were stimulated with PMA, they showed diffuse “clouds” emanating from the disintegrating cells and consisting of DNA with associated eosinophil granule proteins, hallmarks of EETosis (Figure 5A). In contrast, in wells where cells were stimulated with crosslinked CD95, the cells showed altered morphology with sharply demarcated smaller structures, some of which contained DNA, while others contained granule protein MBP. Quantification of DNA and MBP colocalization confirmed our descriptive impression (while no colocalization was seen with NT and IL-5, 3.3 ± 4.7%, and 47 ± 13% of cells showed colocalization with cCD95 and PMA, respectively; *p* < 0.001 PMA compared with NT, IL-5, and cCD95).

Immunofluorescence also enabled us to assess for citrullination of histones (e.g., citrullinated histone 3, citH3), a feature associated with regulated necrosis, in particular EETosis [35]. While only rare populations (<4/HPF) were positive for citH3 in IL5 treatment, there was a marginal increase in citH3 with IL5 withdrawal (NT), and a more profound increase with cCD95 and PMA (Figure 5A,B). Levels of citH3 in LPS alone and LPS/zVAD-treated cells were comparable to NT at 18 h time points (Figure 5A,B). Interestingly, the pattern of citH3 staining mirrored that of DNA. CitH3 positive cell were classified into three distinct sizes: “small”, representing compact puncta; “big”, indicating larger structures (up to ~single cell size); and “net”, characterized by diffuse or web-like spreading across the cell and/or into extracellular space. Quantification revealed that >60% of citH3 positive cells displayed small structures with cCD95, and >60% showed big and net-like structures under PMA stimulation conditions (Figure 5C). Additionally, the size proportion of citH3 in response to LPS/zVAD was no different from untreated cells (Figure 5C).

In summary, we have shown that mouse eosinophils undergo at least three morphologically distinct patterns of cell death (Table 1). While one cell death pattern shows the release of EETs that contain DNA and eosinophil granule proteins (exemplified by PMA stimulation), in the other, cell contents (including DNA and EGP) are organized in distinct small structures (exemplified by cCD95 stimulation). Both types of cell death were associated with citrullination of histones, albeit in a different pattern. In contrast, when cells are activated by LPS/zVAD, there was no increase in citH3 beyond that seen in untreated cells.

### 3.4. Activation Pathways of Eosinophils Associated with Cell Death

**Figure 6:**
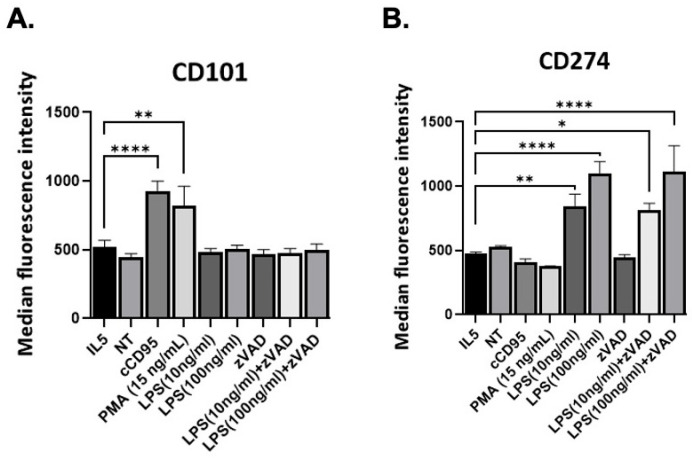
Surface expression of markers of eosinophil activation. BMDeos were incubated with IL5 (10 ng/mL), anti-CD95 crosslinked with protein-A/G (100 ng/mL and 2 μg/mL respectively), PMA (15 ng/mL), LPS (10 and 100 ng/mL) with and without zVAD (20 μM), or no treatment (NT) for 18 h. Level of CD101 (**A**) and CD274 (**B**) was determined on live (7AAD-negative) eosinophils. Data shown are mean ± SEM from 2–4 independent experiments. Statistical analysis by one-way ANOVA followed by Holm–Sidak’s multiple comparison test. * *p* < 0.05, ** *p* < 0.01, **** *p* < 0.0001.

Eosinophil activation is tightly linked to their survival and death pathways. Indeed, many of the same stimuli that drive eosinophil activation can also initiate cell death pathways, depending on the intensity and duration of signaling [19,23,36]. Recent studies have shown two distinct activation/polarization states of eosinophils [37,38]. We tested the hypothesis that eosinophil polarization states are associated with distinct modes of cell death. We assessed the expression of surface activation markers CD274 and CD101, which have been identified as biomarkers distinguishing type 1 and type 2 activated eosinophils, respectively. Both cCD95 and PMA showed increased expression of CD101. However, cells stimulated with LPS (in the presence or absence of zVAD) did not show increased expression of CD101. In contrast, CD274 was not increased by either cCD95 or PMA. LPS did lead to increased CD274 level in cells treated with LPS, and the level was no different irrespective of zVAD (Figure 6A,B). Our results show that LPS and LPS/zVAD are associated with type 1 activation, while cCD95 and PMA are associated with type 2 activation of eosinophils.

## 4. Discussion

In this study, we investigated mouse eosinophil cell death and activation induced by multiple stimuli, and characterized the responses using morphological features and biochemical markers.

Flow cytometry analysis using Annexin V and 7AAD enabled high-throughput quantification of phosphatidyl serine externalization and membrane integrity loss, hallmarks associated with early apoptosis and late-stage apoptosis or necrosis, respectively. Notably, flow cytometry demonstrated greater sensitivity and quantitative resolution in detecting early “apoptotic” events compared to cytologic assessment [39]. However, its analytical capacity is limited to cells in suspension, thus excluding adherent or matrix-bound death modalities such as eosinophil extracellular trap cell death (EETosis), which requires intact substrate contact for execution and visualization [22]. Furthermore, flow cytometry excludes cell aggregates, and studies have shown that certain eosinophil activators (including PMA) induce homotypic aggregation [33,34]. Consequently, cell death patterns that depend on structural context may be underestimated in flow-based assessments, and additional methods should be used for evaluation. On the other hand, cytomorphology alone is not sensitive enough; for instance, IL-5 withdrawal led to cell death through an “apoptosis” pathway detectable by flow cytometry for annexinV/7AAD, with insufficient dying/dead cells recognizable by cytomoprohlogy for accurate assessment. However, together, AnnexinV/7AAD pattern at early time points and cytomorphology by light microscopy were able to distinguish apoptosis (exemplified by cCD95) and regulated necrosis (exemplified by PMA). However, to further subtype the regulated necrosis, additional outcomes were needed.

Caspase-3 cleavage as a surrogate of activation can be measured by flow cytometry and Western blotting. While both modalities showed activation of caspase-3 by both cCD95 and PMA, the timing and extent differed between the two methods. This likely relates to flow cytometry analysis focusing only on intact individual cells (gated on FSC-SSC and doublet exclusion), while Western blotting assesses protein from all cells in the well (including those adhered to surface, aggregates of cells, and dying/dead cells that are no longer intact). Importantly, while traditionally viewed as a hallmark of apoptosis, caspase-3 activation has also been implicated in alternative cell death pathways such as pyroptosis and immunogenic forms of necrosis [17]. Indeed, in our study, caspase-3 was activated by both cCD95 and PMA at a comparable time and level, and as such was unable to discriminate between apoptosis and regulated necrosis.

Human eosinophils undergo a unique cell death subroutine called EETosis [21,22], which is characterized by cytolysis leading to the release of cell-free DNA (cfDNA) and associated cell-free granules/granule proteins. Furthermore, this type of cell death is regulated in part by citrullination of histones, such as citH3 [35]. Identifying this cell death type is important, as levels of cfDNA and citH3 are being investigated as biomarkers of cell necrosis and tissue damage in human disease [40,41,42,43]. Since there is currently no established single gold standard for accurate characterization of EETosis, we measured several outcomes traditionally associated with EETosis. We observed the presence of cfDNA in the supernatant of cells stimulated with both PMA and cCD95 starting at 18 h post-stimulation. While cfDNA is often utilized as a necrotic biomarker, its detection in vitro when pure eosinophil cultures are used may also reflect secondary necrosis of apoptotic cells in the absence of efferocytosis [44,45]. This emphasizes the importance of considering the experimental microenvironment when evaluating cfDNA levels. We thus used immunofluorescent microscopy to assess morphological features including DNA, granule proteins, and citH3, which are essential for distinguishing EETosis from apoptosis or other regulated necrosis [17]. Indeed, PMA stimulated cells showed disintegration of cells with release of “clouds” consisting of DNA and associated eosinophil granule proteins, hallmarks of EETosis, which were not seen in cCD95-stimulated cells. The total level of citH3 was increased in both PMA and cCD95-stimualted cells, consistent with prior reports indicating that citH3 generation can occur during nuclear condensation in apoptosis [46]. However, the morphology of citH3 was different between PMA and cCD95-stimulated cells, in that citH3 was in small distinct puncta in cells stimulated with cCD95, while it was in larger cells and “clouds” when cells were stimulated with PMA. Thus, immunofluorescence staining for DNA/granule proteins and/or citH3 pattern was able to characterize the regulated necrosis (induced by PMA) as EETosis and distinguish it from apoptosis (induced by cCD95).

In conclusion, our data underscore the central tenet that no single measured outcome sufficiently defines cell death identity. Flow cytometry offers sensitivity and quantification; microscopy provides critical morphological context; Western blotting and immunoassays reveal molecular intermediates. Yet each method, on its own, lacks the holistic capacity to delineate the full spectrum of cell death pathways. However, understanding the diversity of cell death forms has profound implications beyond in vitro settings. It is the distinction between homeostatic apoptosis and pathological or inflammatory cell death that influences immune tolerance, cancer surveillance, and host–pathogen dynamics [47]. Thus, accurately classifying death phenotypes as well as its inducers will serve as a framework in identifying how dying cells including eosinophils determine their role, whether physiologically beneficial or pathologically disruptive.

Having identified which outcomes are able to discriminate between apoptosis and regulated necrosis in mouse eosinophils, we assessed eosinophil cell death in response to LPS because eosinophils express PAMP receptors and are exposed to their ligands in inflammatory situations [48]. It has been reported that necroptosis can be achieved by PAMP signaling along with inhibition of caspases [49]; thus, we assessed the effects of LPS in both the presence and absence of zVAD, a pan-caspase inhibitor. LPS alone acted as an eosinophil-survival factor. In contrast, LPS/zVAD induced cell death, which differed from the one induced by both cCD95 and PMA, in that loss of cell viability and release of cfDNA were delayed, there was minimal activation of caspase 3, and there was no increase in citH3. Together, this suggests a different cell death type and pathway; future studies will focus on identifying critical executioners of this distinct cell death type. Specifically, the role of critical molecular executioners of individual cell death types, such as necroptosis, pyroptosis, and ferroptosis, will be tested using inhibitors of those pathways, in parallel with assessing the morphologic, biochemical, and functional outcomes associated with them.

Finally, upon activation, eosinophils undergo functional changes that prime them for effector roles; including degranulation, cytokine secretion, and interaction with other immune cells [50]. However, prolonged activation often triggers programmed cell death (apoptosis or regulated necrosis), serving as a feedback mechanism to prevent tissue injury and maintain immune homeostasis. Indeed, many of the same stimuli that drive eosinophil activation can also initiate cell death pathways, depending on the intensity and duration of signaling [19,36]. Recent studies have shown eosinophil heterogeneity in different disease models and with in vitro activation. Specifically, type 1 eosinophils are activated in vitro with IFNγ and/or bacterial products, are present in the gastrointestinal tract at baseline, and expand with models of bacterial infection or chemical colitis [37,51]. In contrast, type 2 eosinophils are activated in vitro by IL-4, and are prominent in the lung in models of allergic inflammation [37,38]. While different studies use different names and different markers of the activation states, surface expression of CD274 and CD101 are emerging as the most consistent markers of type 1 and type 2 activation, respectively. In our studies, we found that the type 2 activation state can be associated with two different types of cell death (specifically, apoptosis and regulated necrosis). In contrast, type 1 activation is independent of cell death, as it occurred by both LPS alone, which promotes survival, and LPS/zVAD, which causes cell death. Thus, activation state and cell death are not tightly linked.

The limitations of this study include a focus on mouse eosinophils, lack of in vivo confirmation, and lack of functional outcomes. We elected to focus on mouse eosinophils in this study because less is known about eosinophil cell death in mouse eosinophils, and because findings could be used to assess for eosinophil cell death in models of disease in vivo. With findings from this study as baseline, future studies will focus on expanding to human eosinophils, as well as in vivo studies in mouse models and human tissue samples. Furthermore, the identified eosinophil cell death modalities will be tested for their effect on other cell types, such as other immune cells, epithelial cells, or cardiomyocytes, cells that interact with activated and dying eosinophils in vivo.

## 5. Conclusions

In summary, our findings reveal that distinct agents elicit specific patterns of cell death, including apoptosis and regulated necrosis. Importantly, some of the features overlapped and were thus not sufficient to define cell death type. These observations emphasize the complexity of cell death pathways and highlight the need for multi-dimensional approaches in distinguishing cell fates. Furthermore, our study shows that while activation and cell death are often elicited by same stimuli, there is no unique link between subtypes of cell death and specific activation states.

## Figures and Tables

**Table 1:** **Summary** **of outcomes.**

	NT (IL5 Withdrawal)	cCD95	PMA	LPS/zVAD
**Viability by flow**	Small decrease	Big decrease	Big decrease	Decreased viability; delayed compared with cCD95 and PMA
**“Apoptosis”/”necrosis” by flow (at time point when cell death is evident, 4–7 h for NT, cCD95 and PMA; 36 h for LPS/zVAD)**	“Apoptosis” first	“Apoptosis” first	“Necrosis” first	“Apoptosis” first
**Cytospin morphology**	No change	Apoptotic early	Aggregation early, necrotic late; including cell degeneration (both may be underreported by flow)	Vacuolization (seen in LPS alone as well); nuclear pyknosis and budding in LPS/zVAD
**Active caspase flow**	Small increase at both time points; 5 = 18 h	Big increase at both time points; 5 = 18 h	Big increase at both time points; 5 = 18 h	Small increase only at 18 h
**Active caspase Western blot**	No significant increase	5 > 18 h (neither is statistically significant)	5 > 18 h (higher dose statistically sign)	No significant increase
**cfDNA release**	Not statistically significant	Yes (18 h)	Yes (18 h)	Yes (36 h and onward)
**MBP/DNA by IF** **(Normal pattern: BF shows intact cells, DAPI nucleus, MBP cytoplasm, basal level for citH3;** **Apoptotic pattern: cell no longer intact or intact with apoptotic bodies- well demarcated “organelles” containing DNA and/or MBP;** **EET pattern: not well-demarcated clouds/comets/nets etc., containing DNA and MBP)**	Normal	Apoptotic	EET	Normal
**citH3- amount**	Increased, slightly	Increased, very	Increased, intermediate	Increased, slightly
**citH3- pattern**	Mostly small	Mostly small	Mostly big + net	Mostly small
**Description/type**	Intrinsic apoptosis	Extrinsic apoptosis	Regulated necrosis/EETosis	Undefined

## Data Availability

The original contributions presented in this study are included in the article/Supplementary Material. Further inquiries can be directed to the corresponding authors.

## Supplementary Materials

The following supporting information can be downloaded at: https://www.mdpi.com/article/10.3390/cells15060490/s1, Figure S1. Immunofluorescence quantification parameters on PMA treated BMDeos. Supplementary Methods: Minimum Information about a Flow Cytometry Experiment (MIflowcyt).

## Abbreviations

The following abbreviations are used in this manuscript:

cCD95	Crosslinked CD95
BMDeos	Bone marrow-derived eosinophils
PAMP	Pathogen-associated molecular pattern
EAD	Eosinophil-associated diseases
EGID	Eosinophilic gastrointestinal disorders
HES	Hypereosinophilic syndromes
EoE	Eosinophilic esophagitis
NT	No treatment
citH3	Citrullinated histone 3
EGP	Eosinophil granule protein
EETosis	Eosinophil extracellular trap cell death
cfDNA	Cell-free DNA
MBP	Major basic protein
