# Supplementary material for: Activation and Cell Death of Mouse Eosinophils in Response to Different Microenvironmental Stimuli
Source: Cells. 2026 Mar 10;15(6):490. doi: 10.3390/cells15060490 (PMC13024791; doi:10.3390/cells15060490)
Supplement: Supplementary file 1 [file cells-15-00490-s001.zip › cells-4145046-supplementary.pdf]

Supplementary files

for Osuji I and Zimmermann N "Activation and Cell-death of Mouse Eosinophils in Response to Different Microenvironmental Stimuli"

Content:

1. Supplementary Figure S1.

2. Supplementary Methods: Minimum Information about a Flow Cytometry Experiment (MIflowcyt)

Supplementary Figure S1.

Supplemental Figure S1. **Immunofluorescence quantification parameters on PMA treated BMDeos.** Slide wells were imaged using an inverted, motorized Nikon TiE widefield microscope. Slide images were taken with a 40X objective. Marker (citH3) expressions were automatically quantified using the NIS-Element image analysis software (Nikon). **A.** citH3 classification and quantification parameter set up in NIS-element using Boolean operation analysis. Total citH3 as having citH3 staining with sizes ranging from 1.85-414.27um and growing objects. “small” citH3 is having size 1.85-5.50 um, “big” citH3 have sizes of 5.51-12.29um and “net” citH3 have sizes of 12.30-414.27um and growing objects. **B.** Zoomed in representative image of the different classification.

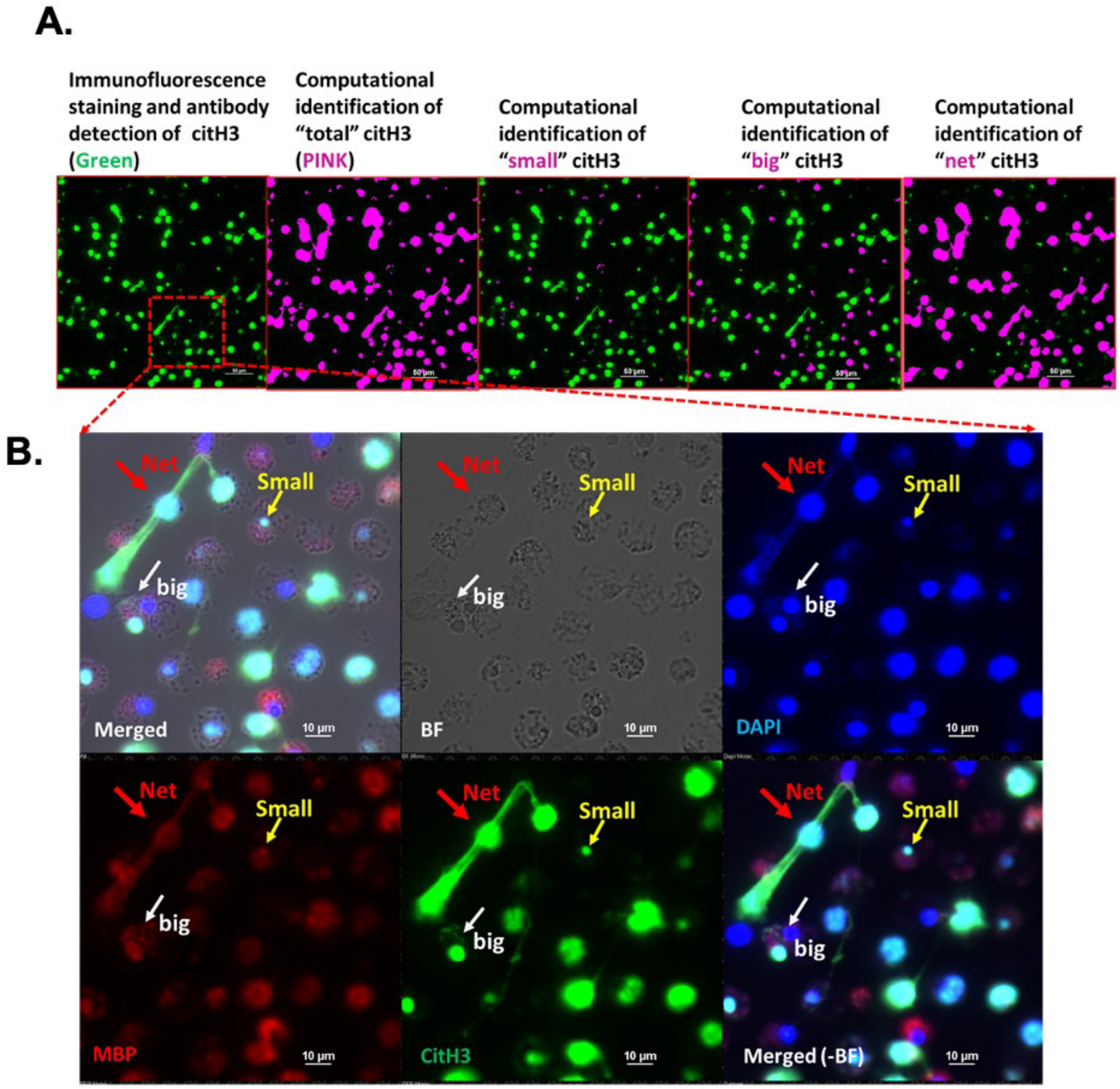

Supplementary methods

Minimum Information about a Flow Cytometry Experiment (MIflowcyt)

Introduction

Description of flow cytometry method used in the BMDeos experiments in compliance with the Minimum Information about a Flow Cytometry Experiment (MIflowcyt). This section describes the MIflowcyt format for visualization and understanding of data interpretation by providing the necessary information on experimental setup and analysis.

1. Experiment Overview

1.1. Purpose

A. Cell culture: to propagate cells under controlled in-vitro conditions, enabling experimental manipulation and assessment of cellular response to defined stimuli or treatment.
B. Cell death: to quantify and distinguish viable, early apoptotic and late apoptotic/necrotic cells based on phosphatidylserine externalization and plasma membrane integrity.
C. Caspase 3 activation: To assess activation of the executioner caspase-3 as a marker of apoptosis.
D. Cell activation: To evaluate cellular activation status by measuring the expression of activation-associated surface markers in response to stimulation or treatment.

1.2. Keywords

Bone marrow cells, eosinophils, cell death

1.3. Experiment Variables

A. Cell culture
Primary mouse eosinophils were differentiated *in vitro* from bone marrow derived cells. Their maturation was monitored by expression of surface eosinophil markers.
B. Cell death
Cells were divided into treatment groups receiving indicated stimulus and a vehicle control group. All conditions were assessed after a fixed incubation period, with equal cell numbers plated per condition. Following treatment, eosinophils from each condition were stained with Annexin-V and 7AAD. For each treatment group, the percentages of Annexin V^{-}/7-AAD^{-} (viable), Annexin V^{+}/7-AAD^{-} (early apoptotic), and Annexin V^{+}/7-AAD^{+} (late apoptotic/necrotic) cells were quantified by flow cytometry. Identical staining conditions were applied across all groups.
C. Caspase3 activation
Eosinophils exposed to the same treatment conditions were fixed, permeabilized, and stained for activated caspase3. The proportion of caspase3 positive cells were measured for each treatment group.

D. Cell activation
Eosinophils were stimulated with the indicated activator or unstimulated controls. Expression of activation-associated surface markers was assessed by flow cytometry, and the Median fluorescence intensity was determined for each experimental group.

1.4. Organization

1.4.1. Name: University of Cincinnati College of Medicine, Department of Pathology and Laboratory Medicine and Department of Pharmacology, Physiology and Neurobiology

1.4.2. Address: 3230 Eden Ave, Cincinnati, OH 45267; USA

1.5. Primary Contact

1.5.1. Name: Nives Zimmermann
1.5.2. Email: zimmerns@ucmail.uc.edu

1.6. Date

A. Cell culture_Multiple *in vitro* experiments were performed between April 2022 and November 2025.
B. Cell death_Multiple *in vitro* experiments were performed between April 2022 and February 2023.
C. Caspase3 activation_Multiple *in vitro* experiments were performed between September 2022 and March 2024.
D. Cell activation_Multiple *in vitro* experiments were performed between October 2024 and May 2025

1.7. Conclusions: See manuscript

1.8. Quality Control Measures

Unstimulated and stimulated BMDeos were used for compensation controls, gating and FMO.

1.9. Other Relevant Experiment Information: N/A

2. Flow Sample/Specimen Details

2.1. Sample/Specimen Material Description

2.1.1. Biological Samples
2.1.1.1. Biological Sample Description: bone marrow derived cells.
2.1.1.2. Biological Sample Source Description: Mus musculus, BALB/c background
2.1.1.3. Biological Sample Source Organism Description:

- Taxonomy: Mus musculus, BALB/c background
- Age: >6wk-old mice
- Gender: Female
- Phenotype: white
- Genotype: wild type

- Stimulation: BMDeos with LPS (10, 50, 100ng/ml), IL-5 (10ng/ml), zVAD (20uM), protein A/G (2ug/ml), CD95 (100ng/ml) and CD95 crosslinked with protein A/G (cCD95)

2.1.2. Environmental Samples: N/A

2.1.3. Other Samples: N/A

2.2. Sample Characteristics

BMDeos are a pure cell population.

2.3. Sample Treatment Description

A. Cell culture

- bone marrow cells were flushed from mouse femurs and tibiae using warm culture media.
- Pelleted, and red blood cells lysed with lysis buffer for 2min at RT
- Reaction was quenched with 1x dPBS and centrifuged at 400g for 15min
- Pellet was resuspended in culture media at density of 1x10^6/ml
- Media was fed SCF and FLT-3L for 4 days after which cells were washed with cytokine free media and replaced with fresh media supplemented with IL5 every other day.

B. Cell death

- After treatment, BMDeos were collected and washed with FACS buffer
- Fc receptors were blocked using anti-mouse CD16/CD32 antibody (Invitrogen eBioscience) for 10 minutes on ice in FACS buffer.
- Cells were incubated with antibodies (or controls) for 30 minutes at 4°C in staining buffer (approx. 10^6 cells in 200µl of staining buffer).
- After the incubation, cells were washed 1x with 1ml of FACS buffer and pelleted by centrifugation (300g for 5 min); supernatant has been removed.
- Finally, cells were resuspended in 400ul Annexin-v buffer with 7AAD and Annexin-V, incubated at room temperature for 15min prior to flow analysis

C. Activation of Caspase 3

- After treatment, cells were washed twice with cold 1X PBS, then resuspend cells in BD Cytofix/Cytoperm solution at a concentration of 1x10^6 cells/0.5 ml.
- Incubated for 20 min on ice.
- Pellet cells, aspirate, and discard BD Cytofix/Cytoperm solution
- washed twice with BD Perm/Wash buffer (1X) at a volume of 0.5 ml buffer/1x10^6 cells at room temperature.
- Resuspended in BD Perm/Wash buffer (1X) plus antibody and incubate for 30 min at room temperature.
- Washed with Perm/Wash buffer (1X)
- then resuspend in 0.4 ml BD Perm/Wash buffer (1X) and analyze by flow cytometry

D. Cell activation

- After treatment, BMDeos were collected and washed with FACS buffer
- Fc receptors were blocked using anti-mouse CD16/CD32 antibody (Invitrogen eBioscience) for 10 minutes on ice in FACS buffer.

- Cells were incubated with antibodies (or controls) for 30 minutes at 4°C in staining buffer (approx. 10^6 cells in 200µl of staining buffer).
- After the incubation, cells were washed 1x with 1ml of FACS buffer and pelleted by centrifugation (300g for 5 min); supernatant has been removed.
- Finally, cells were resuspended in 400ul FACS buffer with 7AAD and incubated at room temperature for 15min prior to flow analysis

2.4. Fluorescence Reagent Description

Compensation tubes have been created as follows:

Single staining									
Reporter	PE	APC	FITC	BV421	PEcy7	APCcy7	BV605	7AAD	none
Tube#1	+Ab	-	-	-	-	-	-	-	-
Tube#2	-	+Ab	-	-	-	-	-	-	-
Tube#3	-	-	+Ab	-	-	-	-	-	-
Tube#4	-	-	-	+Ab	-	-	-	-	-
Tube#5	-	-	-	-	+Ab	-	-	-	-
Tube#6	-	-	-	-	-	+Ab	-	-	-
Tube#7	-	-	-	-	-	-	+Ab	-	-
Tube#8	-	-	-	-	-	-	-	7AAD	-
Tube#9	-	-	-	-	-	-	-	-	-

FMOs								
Reporter	PE	BV421	APCcy7	APC	FITC	PEcy7	BV605	7AAD
FMO-APC	+Ab	+Ab	+Ab	-	+Ab	+Ab	+Ab	+Ab
FMO-FITC	+Ab	+Ab	+Ab	+Ab	-	+Ab	+Ab	+Ab
FMO-PEcy7	+Ab	+Ab	+Ab	+Ab	+Ab	-	+Ab	+Ab
FMO-BV605	+Ab	+Ab	+Ab	+Ab	+Ab	+Ab	-	+Ab
FMO-7AAD	+Ab	+Ab	+Ab	+Ab	+Ab	+Ab	+Ab	-

Each sample has been stained as follows:

Reporter	PE	APC	FITC	BV421	PEcy7	APCcy7	BV605	7AAD
Sample	+Ab	+Ab	+Ab	+Ab	+Ab	+Ab	+Ab	7AAD

A. cell culture

Analyte	Reporter	Detector	dilution	Target	Manufacturer	Cat#
Siglec-F	PE	CD170 (Siglec F) Antibody	1:100	eosinophils	Invitrogen	155506
CCR3	BV421	anti-mouse CD193	1:100	eosinophils	Biolegend	cat# 144517

		(CCR3) Antibody				
viability	7AAD		1:100	Live/dead	eBioscience	006993-50
CD45	APCcy7	Rat Anti-Mouse CD45 Antibody	1:100	Hematopoietic cells	BD Pharmingen	1046748

B. Cell death

**Analyte**	**Reporter**	**Detector**	**Dilution**	**Target**	**Manufacturer**	**Cat#**
Siglec-F	PE	CD170 (Siglec F) Antibody	1:100	eosinophils	Invitrogen	155506
CCR3	BV421	anti-mouse CD193 (CCR3) Antibody	1:100	eosinophil	Biolegend	cat# 144517
viability	7AAD		1:100	Live/dead	eBioscience	006993-50
Annexin-v	BV605		1:100	Live/dead		

C. caspase 3 activation

**Analyte**	**Reporter**	**Detector**	**dilution**	**Target**	**Manufacturer**	**Cat#**
Active Caspase 3	FITC		20ul	Intracellular activated caspase 3	BD Pharmingen	50480

D. cell activation

**Analyte**	**Reporter**	**Detector**	**Dilution**	**Target**	**Manufacturer**	**Cat#**
Siglec-F	PE	CD170 (Siglec F) Antibody	1:100	eosinophil	Invitrogen	155506
CCR3	BV421	anti-mouse CD193 (CCR3) Antibody	1:100	eosinophil	Biolegend	cat# 144517
viability	7AAD		1:100	Live/dead	eBioscience	006993-50
CD274	APC	Anti-mouse CD274 (B7-H1,	1:100	Cell activation	Biolegend	124311

		PD-L1) Antibody				
CD101	Alexa Fluor700	Anti-mouse CD101 Monoclonal Antibody	1:100	Cell activation	eBioscience	56-1011-82

3. instrument Details

3.1. Instrument Manufacturer

BD Beckton Dickinson

3.2. Instrument Model

BD/FACSCanto
BD/LSRFortessa

Technical specification at: https://www.cincinnatichildrens.org/research/cores/flow-cytometry

3.3. Instrument Configuration and Settings

3.3.1. Flow Cell and Fluidics

The instruments have not been altered

3.3.2. Light Sources

The instruments have not been altered

3.3.3. Excitation Optics Configuration

The instruments have not been altered.

**Number of Fluorescence PMTs**						
**Instrument Name***	Instrument Make/Model**	UV (355nm)	Violet (405nm)	Blue (488nm)	Yellow-Green (561nm)	Red (635nm)
**Canto 3**	BD/FACSCanto		3	5		3
**Instrument Name***	**Instrument Make/Model****	**UV (355 nm) 20mW**	**Violet (405 nm) 50mW**	**Blue (488 nm) 60mW)**	**Yellow-Green (561 nm) 50mW**	**Red (640 nm) 40mW**
**Fortessa**	BD/LSRFortessa	2	6	2	5	3

3.3.4. Optical Filters

The instruments have not been altered, all filters are original and came with the instrument.

3.3.5. Optical Detectors

The instruments have not been altered.

3.3.6. Optical Paths

The instruments have not been altered. The following figure shows the filter and detector configuration:

Optical detectors configuration_Cantos:

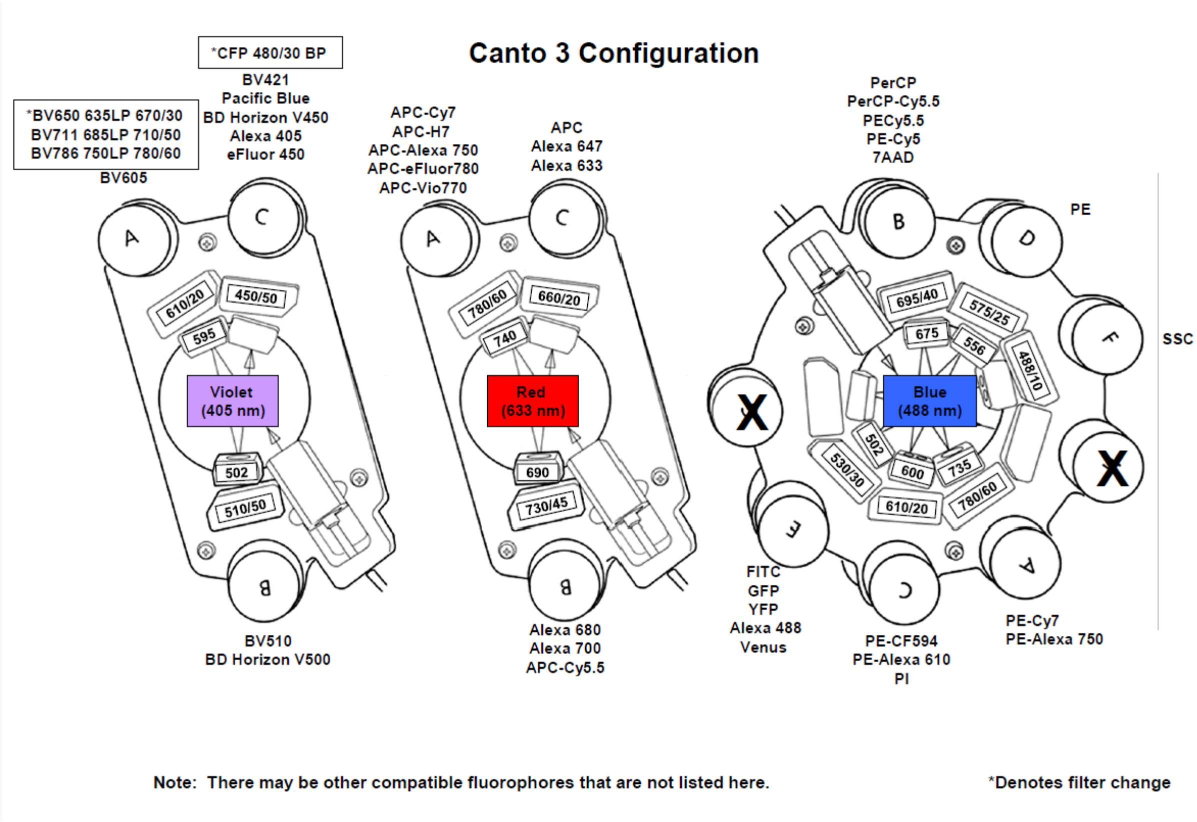

Optical detectors configuration detail_Cantos:

Detector Array (laser)	PMT	LP Mirror	BP filter	Dye/detector
Violet (405nm)	A	595	610/20	635LP 670/30<br>BV711 685LP 710/50<br>BV786 750LP 780/60<br>BV605
	B	502	510/50	BV510<br>BD Horizon V500
	C	-	450/50	BV421<br>Pacific Blue<br>BD Horizon V450<br>Alexa 405<br>eFluor 450<br>*CFP 480/30 BP
Red (633 nm)	A	740	780/60	APC-Cy7<br>APC-H7<br>APC-Alexa 750<br>APC-eFluor780<br>APC-Vio770
	B	690	730/45	Alexa 680<br>Alexa 700<br>APC-Cy5.5
	C	-	660/20	APC<br>Alexa 647<br>Alexa 633
Blue (488nm)	A	735	780/60	PE-Cy7

				PE-Alexa 750
	B	675	695/40	PerCP<br>PerCP-Cy5.5<br>PECy5.5<br>PE-Cy5<br>7AAD
	C	600	610/20	PE-CF594<br>PE-Alexa 610<br>PI
	D	556	575/25	PE
	E	502	530/30	FITC<br>GFP<br>YFP<br>Alexa 488<br>Venus
	F	-	488/10	

Optical detectors configuration_fortessa:

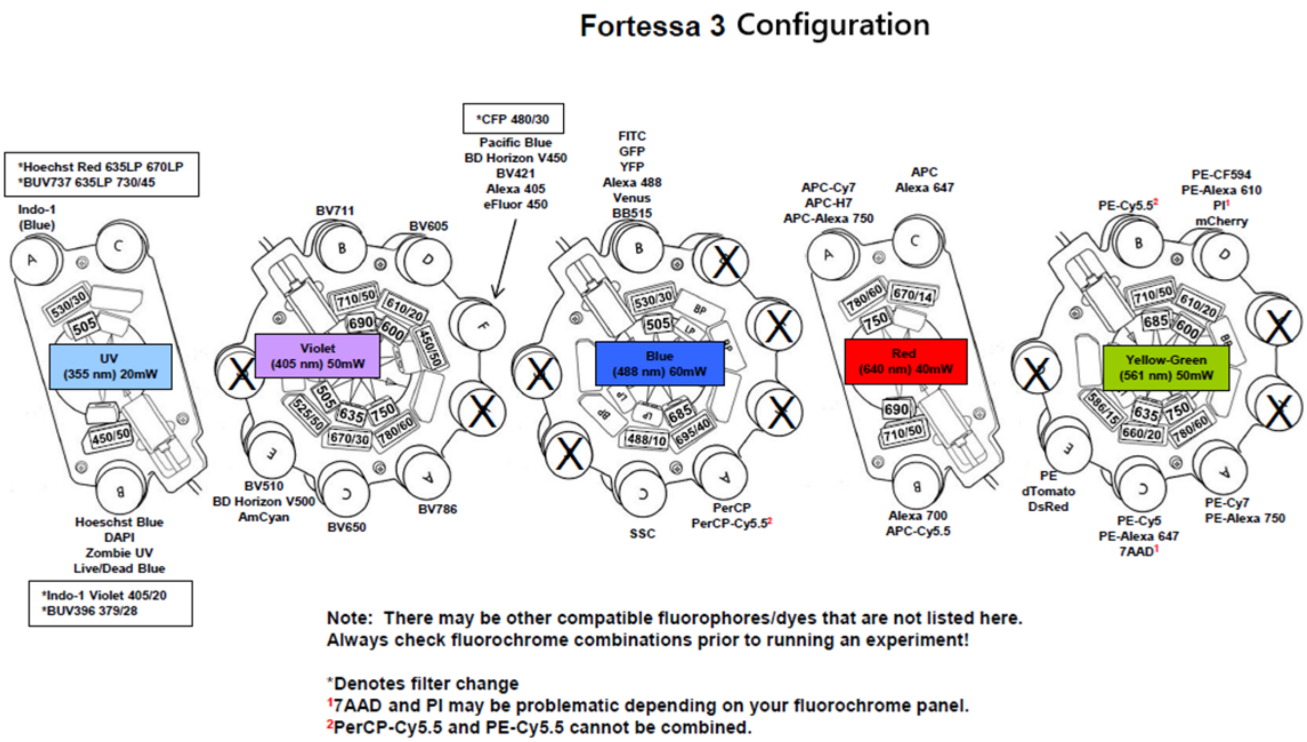

Optical detectors configuration detail_fortessa

Detector Array (laser)	PMT	LP Mirror	BP filter	Dye/detector
UV<br>(355 nm) 20mW	A	505	530/30	Indo-1 (Blue)<br>*Hoechst Red 635LP 670LP<br>*BUV737 635LP 730/45

	B		450/50	HoeschstBlue<br>DAPI<br>Zombie UV<br>Live/Dead Blue<br>*Indo-1 Violet 405/20<br>*BUV396 379/28
	C			
Violet<br>(405 nm) 50mW	A	750	780/60	BV786
	B	690	710/50	BV711
	C	635	670/30	BV650
	D	600	610/20	BV605
	E	505	525/50	BV510<br>BD Horizon V500<br>AmCyan
	F		450/50	Pacific Blue<br>BD Horizon V450<br>BV421<br>Alexa 405<br>eFluor450<br>*CFP 480/30
Blue<br>(488 nm) 60mW	A	685	695/40	PerCP<br>PerCP-Cy5.5[2]
	B	505	530/30	FITC<br>GFP<br>YFP<br>Alexa 488<br>Venus<br>BB515
	C		488/10	SSC
Red<br>(640 nm) 40mW	A	750	780/60	APC-Cy7<br>APC-H7<br>APC-Alexa 750
	B	690	710/50	Alexa 700<br>APC-Cy5.5
	C		670/14	APC<br>Alexa 647
Yellow-Green<br>(561 nm) 50mW	A	750	780/60	PE-Cy7<br>PE-Alexa 750
	B	685	710/50	PE-Cy5.5[2]
	C	635	660/20	PE-Cy5<br>PE-Alexa 647<br>7AAD[1]
	D	600	610/20	PE-CF594<br>PE-Alexa 610<br>PI[1]<br>mCherry
	E		586/15	PE-Cy5.5[2]

3.4. Other Relevant Instrument Details

https://www.cincinnatichildrens.org/research/cores/flow-cytometry/analyzer
https://www.cincinnatichildrens.org/research/cores/flow-cytometry/software

4. Data Analysis Details

4.1. List-mode Data Files

FCS data files can be obtained by contacting Dr. Nives Zimmermann after this work has been published.

4.2. Compensation Description

Compensation has been performed computationally post acquisition according to the following spillover matrix (values in %):

A. Cell culture

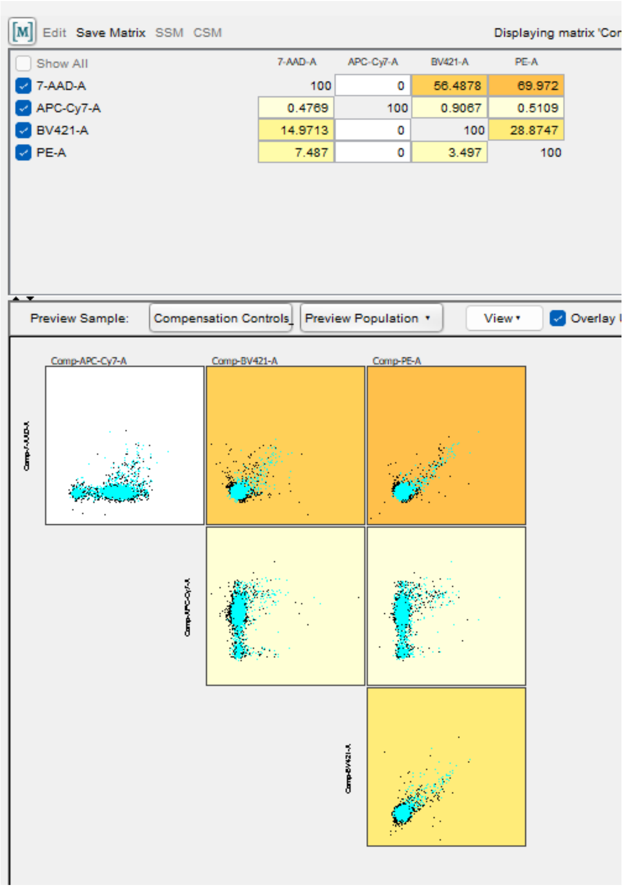

	7-AAD-A	APC-Cy7-A	BV421-A	PE-A
7-AAD-A	100	0	56.4878	69.972
APC-Cy7-A	0.4769	100	0.9067	0.5109
BV421-A	14.9713	0	100	28.8747
PE-A	7.487	0	3.497	100

B. Cell death

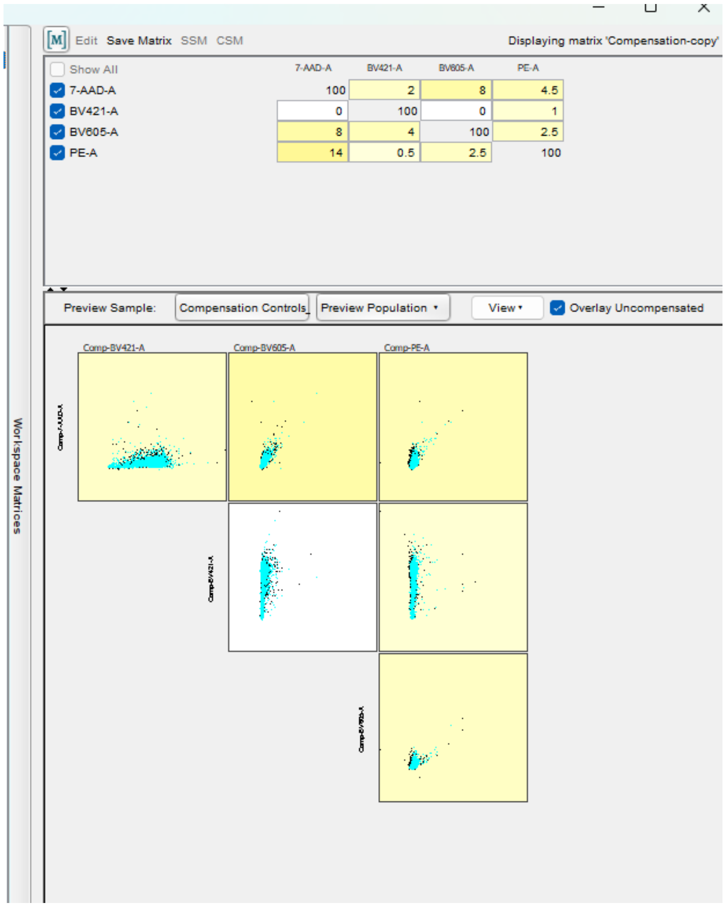

C. Caspase 3 activation

N/A

D. Cell activation

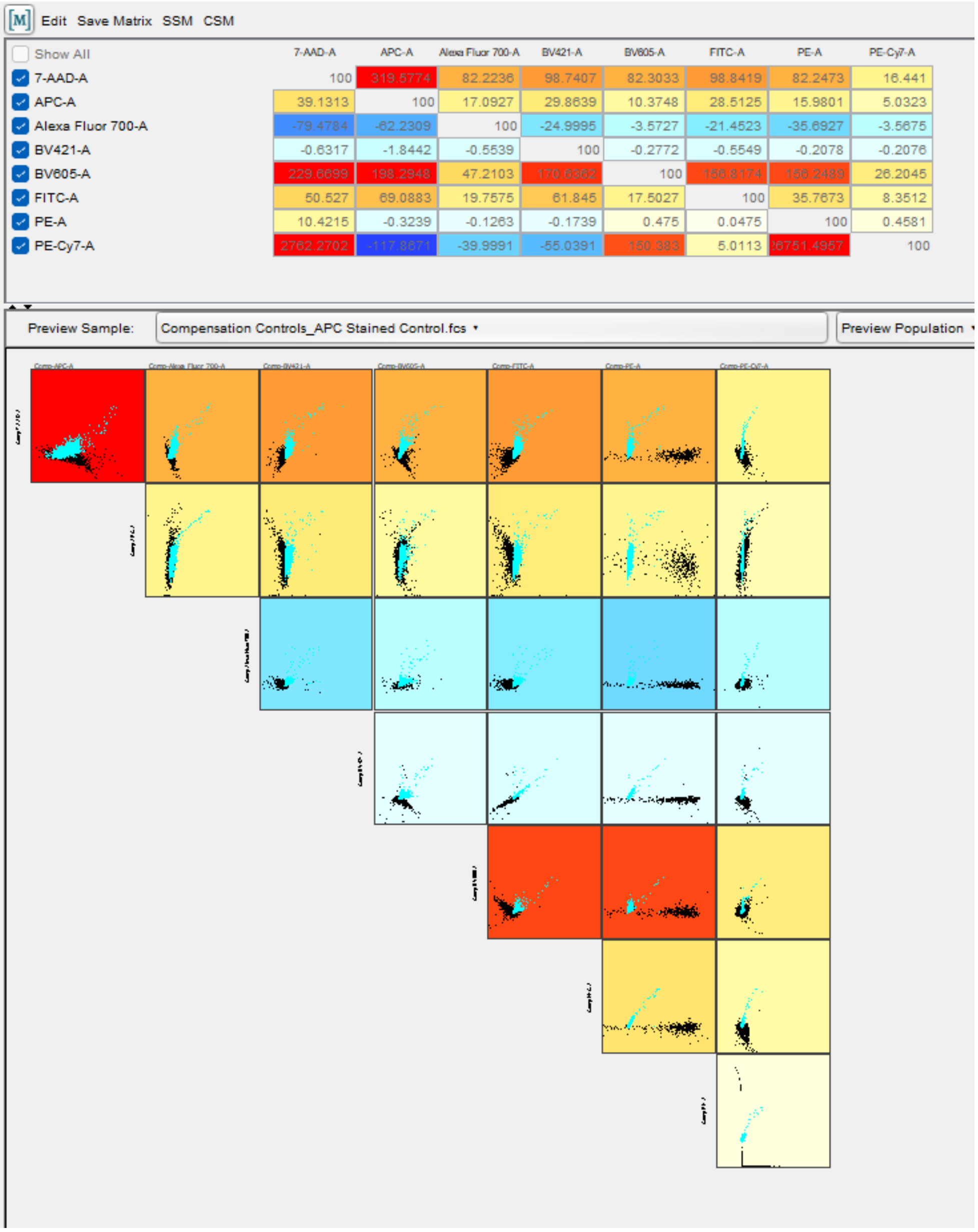

	7-AAD-A	APC-A	Alexa Fluor 700-A	BV421-A	BV605-A	FITC-A	PE-A	PE-Cy7-A
7-AAD-A	100	319.5774	82.2236	98.7407	82.3033	98.8419	82.2473	16.441
APC-A	39.1313	100	17.0927	29.8639	10.3748	28.5125	15.9801	5.0323
Alexa Fluor 700-A	-79.4784	-62.2309	100	-24.9995	-3.5727	-21.4523	-35.6927	-3.5675
BV421-A	-0.6317	-1.8442	-0.5539	100	-0.2772	-0.5549	-0.2078	-0.2076
BV605-A	229.6699	198.2948	47.2103	170.6362	100	156.8174	156.2489	26.2045
FITC-A	50.527	69.0883	19.7575	61.845	17.5027	100	35.7673	8.3512
PE-A	10.4215	-0.3239	-0.1263	-0.1739	0.475	0.0475	100	0.4581
PE-Cy7-A	2762.2702	-117.8671	-39.9991	-55.0391	150.383	5.0113	26751.4957	100

4.3. Data Transformation Details

4.3.1.Purpose of Data Transformation

Visualization and gating.

4.3.2.Data Transformation Description

FlowJo default visualization settings have been used for gating:

4.3.3.Other Relevant Data Transformation Details

- the default visualization setting in flowjo was used for gating:
- When digital data (fcs files) are brought into flowjo it automatically shows biexponentially transformed using logicle implementation.
- Scale: Biex
- Transforms:
  - Extra Negative decades:0

- wideth basis:-100
- Positive decades:4.42

4.4. Gating (Data Filtering) Details

In order to keep this document simple, we provide details only one gating of a single sample and we include these as images within this document.

4.4.1. Gate Description

The gating strategy involves the following gates:

A. Cell culture

- Total cells were gathered by FSC-SSC gating; this was called “bone marrow cells (BMC)”
- From the BMC, “single cells” were selected by applying FSC-A/FSC-H gating (doublet exclusion)
- From “single cells”, dead cells were removed, and the remaining cells are called “7AAD-cells”
- From “live cells”, “CD45 positive” (hematopoietic) cells were selected
- From “CD45+”, we identified Siglec-F+ (sigF), CCR3+ and eos (sigF+CCR3+)

B. Cell death

- Total cells were gathered by FSC-SSC gating this was called bone marrow derived eosinophil (BMDeos)
- From the BMDeos, single cells were selected by applying FSC-A/FSC-H gating and from singles cells
- Eosinophil (Eos) are determined by Siglec-F+/CCR3+
- From Eos population viable cells were assessed by 7AAD and Annexin-V.

C. Caspase 3 activation

- Total cells were gathered by FSC-SSC gating this was called bone marrow derived eosinophil (BMDeos)
- From the BMDeos, single cells were selected by applying FSC-A/FSC-H gating and from singles cells
- From single cells caspase 3 activation identified.

D. Cell activation

- Total cells were gathered by FSC-SSC gating this was called BMDeos
- From the BMDeos, single cells were selected by applying FSC-A/FSC-H gating and from singles cells
- Eosinophil (Eos) are determined by Siglec-F+/CCR3+
- Dead cells were removed by 7AAD, and from viable cells CD101 positive population was determined and MFI of the positive CD101 cell was determined
- Also, from the viable cell MFI of CD274 was assessed.

4.4.2.Gate Statistics

The following table shows an example of percentages of each of the subpopulations defined by described gates.

A. Cell culture

ID# day2 sample_0204	Total bone marrow cells (BMC)	Single cells	Live cells (7AAD-)	CD45+	CCR3+	Siglec-F+	Siglec-F+CCR3+
% of FSC-SSC	89.2						
% of FSC-SSC		99.4					
% of single cells			90.2				
% of live cells				78.6			
% of CD45+					3.62	3.50	0.99

B. Cell death

ID# rmIL5_M1IL1	BMDeos	Single cells	Eos (Siglec-f+CCR3+)	7AAD+	Annexin-v+	7AAD+Annexin-V+	7AAD-Annexin-V-
% of FSC-SSC	91.8						
% of FSC-A/H		99.7					
% of single cells			98.4				
% of Eos				0.22	3.96	2.79	93.1

C. Caspase 3 activation

ID# active Cas3 M1_1	BMDeos	Single cells	Active-Casp3
% of total cell	92.9		
% of BMDeos		99.6	
% of single cells			2.65

D. Cell activation

ID# M1_PA#1	BMDeos	Single cells	Eos (Siglec-f+CCR3+)	Live cells (7AAD-)	CD274	CD101	MFI CD274	MFI CD101
% of FSC-SSC	85.0							
% of FSC-A/H		99.5						
% of single cells			96.1					
% of Eos				86.9				
%of live					6.0	43.1		
Of live							496	
Of CD101								486

Gate Statistics applies to all used data files in a real experiment description. We only provide a single data file in order to keep this document as a clear and simple example.

4.4.3.Gate Boundaries

A. Cell culture

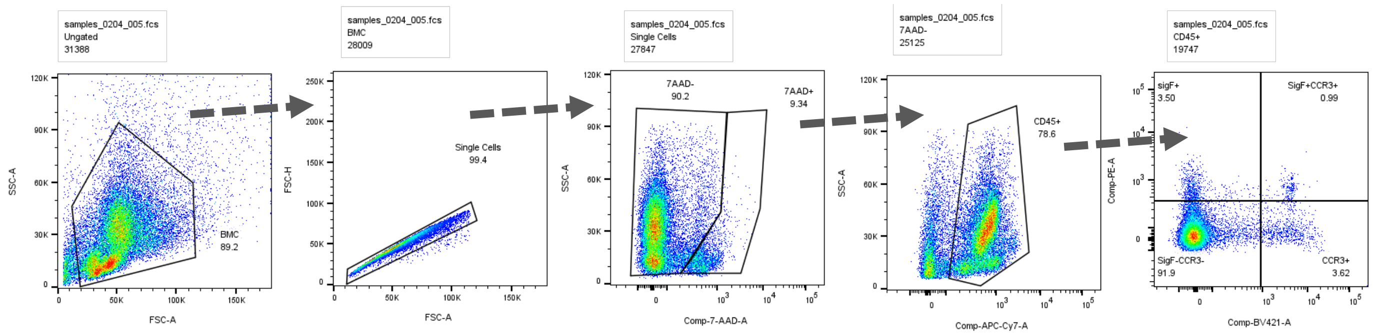

B. Cell death

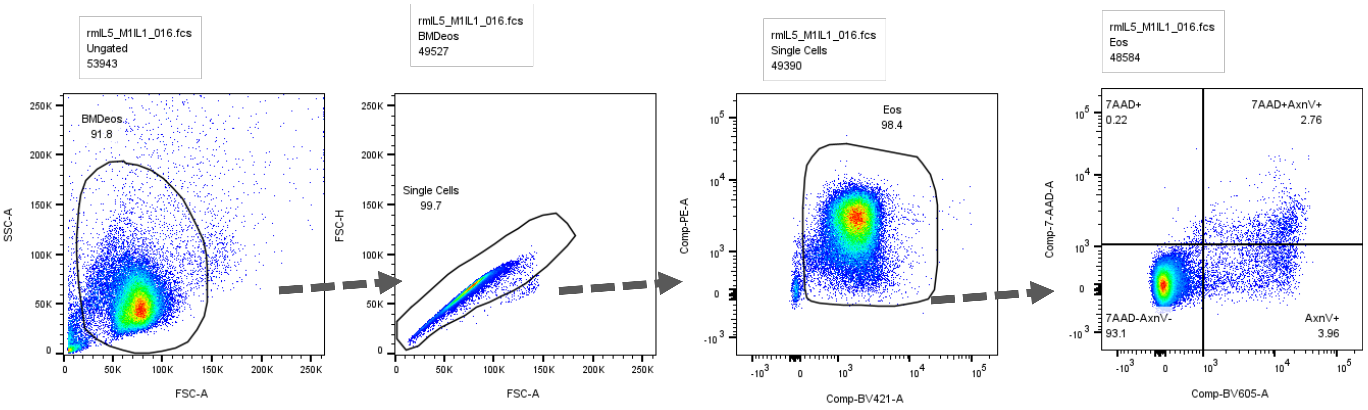

C. Caspase 3 activation

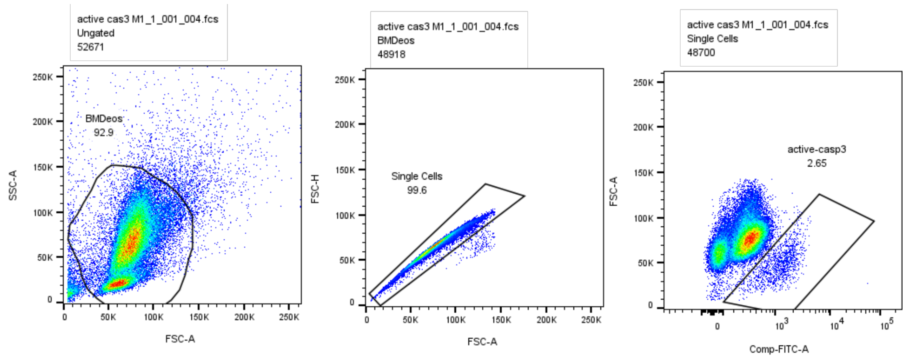

D. Cell activation

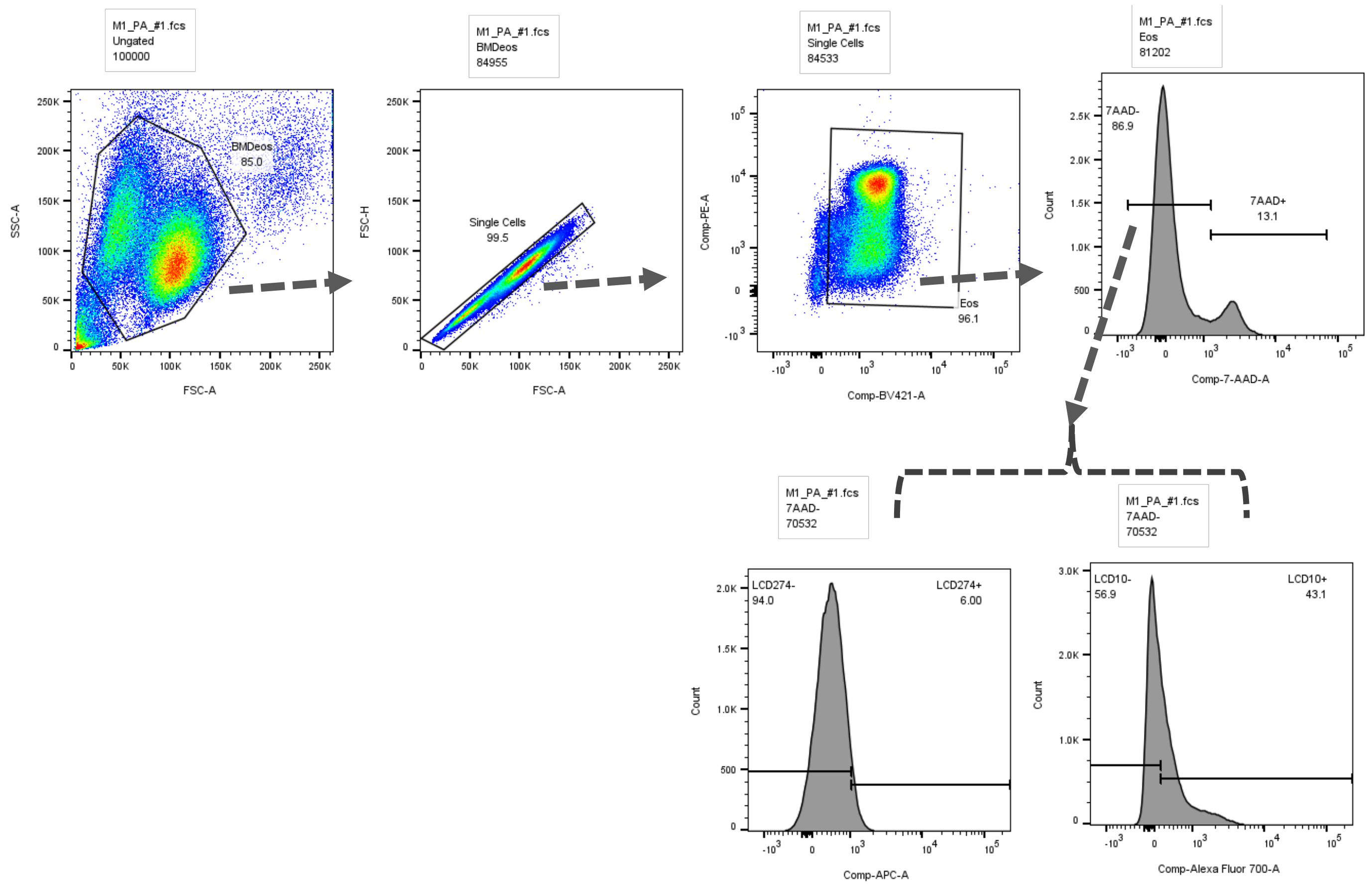

4.4.4. Other Relevant Gate Information

FlowJo workspace files could be obtained by contacting Dr. Nives Zimmermann after this work has been published.
